# Rapid Assessment of Crystal Nucleation and Growth Kinetics: Comparison of Seeded and Unseeded Experiments

**DOI:** 10.1021/acs.cgd.2c01406

**Published:** 2023-06-20

**Authors:** Andrew Cashmore, Russell Miller, Hikaru Jolliffe, Cameron J. Brown, Mei Lee, Mark D. Haw, Jan Sefcik

**Affiliations:** † Department of Chemical and Process Engineering, University of Strathclyde, 75 Montrose Street, Glasgow G1 1XJ, U.K.; ‡ CMAC Future Manufacturing Research Hub, Technology and Innovation Centre, 99 George Street, Glasgow G1 1RD, U.K.; § GlaxoSmithKline, Product Development and Supply, Gunnellswood Rd, Stevenage SG1 2NY, U.K.

## Abstract

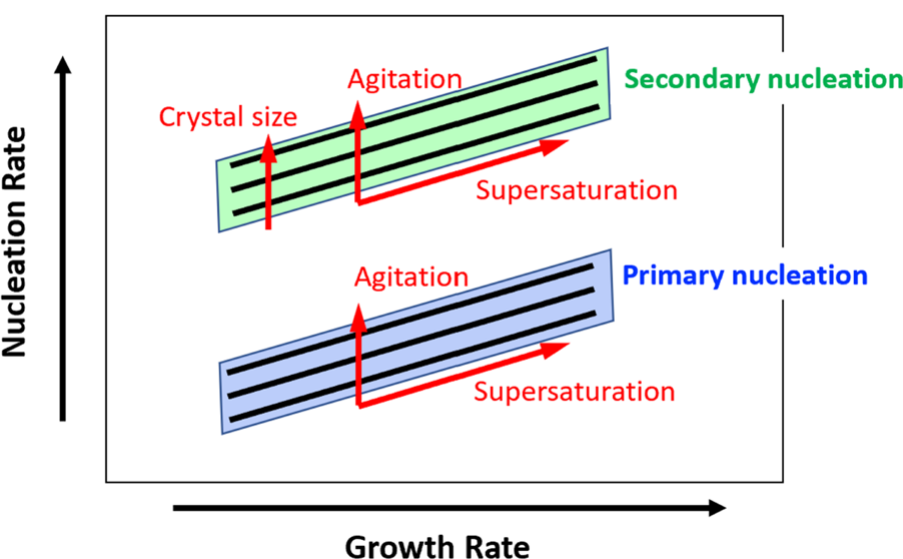

In this work, we outlined an experimental workflow enabling the rapid assessment of primary and secondary nucleation and crystal growth kinetics. We used small-scale experiments in agitated vials with in situ imaging for crystal counting and sizing to quantify nucleation and growth kinetics of α-glycine in aqueous solutions as a function of supersaturation at isothermal conditions. Seeded experiments were required to assess crystallization kinetics when primary nucleation is too slow, especially at lower supersaturations often encountered in continuous crystallization processes. At higher supersaturations, we compared results from seeded and unseeded experiments and carefully analyzed interdependencies of primary and secondary nucleation and growth kinetics. This approach allows for the rapid estimation of absolute values of primary and secondary nucleation and growth rates without relying on any specific assumptions about functional forms of corresponding rate expressions used for estimation approaches based on fitting population balance models. Quantitative relationships between nucleation and growth rates at given conditions provide useful insights into crystallization behavior and can be explored to rationally manipulate crystallization conditions for achieving desirable outcomes in batch or continuous crystallization processes.

## Abstract 2

The table of contents graphic shows the importance of considering what are the effects of processing conditions on primary and secondary nucleation and crystal growth kinetics. First of all, as supersaturation increases, both crystal growth and nucleation rates increase in tandem. An increase in crystal size and agitation rate may then be used to increase the nucleation kinetics.

## Introduction

Crystal nucleation and growth are inexorably linked when manifested in typical observations of crystallization behavior, conspiring together to determine the final particle size and shape distribution. While crystal growth rate measurements have been relatively well established, based on single-crystal growth observations or desupersaturation measurements in seeded crystallization carefully controlled to minimize nucleation, crystal nucleation kinetics measurements have been more challenging.^1^ In order to observe primary nucleation, crystal nuclei must grow to be large and/or numerous enough to be detected and/or to induce secondary nucleation, which rapidly generates further crystals that can then be detected.^2^ There are several different approaches to determine primary or secondary nucleation kinetics, based on non-isothermal metastable zone width measurements,^3,4^ isothermal induction time measurements,^5^ particle counting,^6,7^ or particle sizing approaches.^1^ Each of these approaches relies on certain assumptions about the process and underlying models used to estimate primary or secondary nucleation rates.

In particular, it may be challenging to decouple primary and secondary nucleation effects in agitated systems, where both primary and secondary nucleation are sensitive to agitation conditions^7−9^ and relative magnitudes of primary and secondary nucleation rates are not known a priori. Furthermore, it is important to account for crystal growth kinetics to properly interpret induction times observed under various conditions and decouple the effects of nucleation and growth kinetics.

Crystal growth and nucleation rates generally follow power law dependencies on supersaturation,^10^ and this leads to observations of apparent dead zones^11^ or thresholds^12^ at low supersaturations when kinetic data is plotted using linear scales. However, this is often related to sensitivity limits of experimental methods used, and care must be taken when interpreting and presenting such results. For example, isothermal induction time experiments can be conveniently conducted at timescales from minutes to hours or days, which requires a characteristic time of nucleation 1/*JV* on the comparable timescales, so that only up to three orders of magnitudes of absolute values of primary nucleation rates *J* are practically accessible at a given volume scale *V*.

Systematic workflow approaches to crystallization process development and characterization have been presented in recent literature^7,13,14^ based on a rapid experimental screening using widely available crystallization platforms. These approaches use small-scale experiments to provide data on crystallization kinetics under a wide range of experimental conditions. When such data are carefully analyzed accounting for relevant kinetics interdependencies, corresponding primary and secondary nucleation and growth kinetics can be estimated. This then allows for the assessment of the crystallization behavior in terms of relative magnitudes of nucleation and growth kinetics in relation to relevant characteristic times.^15,16^ It is important to recognize that primary and secondary nucleation can have very different magnitudes^17^ and that changing supersaturation will change both nucleation and growth kinetics and also that nucleation kinetics, in particular, can have a strong dependence on other process parameters such as agitation. It is therefore paramount that underlying kinetic effects are properly quantified and decoupled as highlighted above. Resulting quantitative relationships between nucleation and growth rates at given conditions provide useful insights into crystallization behavior and can facilitate rational manipulation of crystallization conditions for achieving desirable outcomes in batch or continuous crystallization processes.

In this work, we present a systematic workflow based on small-scale experiments in agitated vials to estimate primary and secondary nucleation kinetics as well as crystal growth kinetics under isothermal conditions. In particular, we show that all of these data can be, in principle, obtained from the same crystallization experiment in a single vial. We carefully analyze interdependencies of primary and secondary nucleation and crystal growth kinetics and the corresponding crystallization behavior of α-glycine in aqueous solutions.

## Experimental Section

### Solubility and Metastable Zone Width

In this study, the solubility and metastable zone width were determined using the Crystal 16 (Technobis Crystallization Systems) instrument. This setup enables precise control over the temperature cycling programs of individual vials with built-in optical technology to measure a change in transmissivity identifying the temperature where clear points or cloud points are reached, related to complete dissolution or initial crystal formation, respectively.

Individual vials at a given glycine concentration were prepared by weighing a known mass of finely ground glycine powder directly into the vials (VWR, total volume 1.5 mL). Once deionized water (1 mL) was added, the vials were tightly sealed and placed into the reactor system. The solution was first heated from room temperature to 70 °C to dissolve the solid glycine, and solutions were agitated using a magnetic stirrer bar at 700 rpm. Complete dissolution is indicated by a transmissivity value of 100% for the resulting clear solution. The temperature was then decreased from 70 to 5 °C at a fixed cooling rate (cooling rates were from 0.1 to 0.5 °C/min). As crystallization begins, transmissivity decreases. Once transmissivity decreases below 50%, the corresponding temperature is recorded to indicate the metastable limit under given conditions. Choosing another transmissivity value, such as 10 or 90%, results in similar metastable limit temperatures which are all highly correlated to each other. Following a hold period at 5 °C for 15 min, the sample was heated back to 70 °C at a fixed heating rate (heating rates were from 0.1 to 0.5 °C/min), and a change in transmissivity from 0 to 100% is observed on dissolution of suspended crystals. The temperature at which the transmissivity reaches 100% is taken as the clear point at the given concentration and heating rate. Each cycle was completed three times. Multiple heating rates were selected to account for the effect of the dissolution kinetics on measured clear point values. Clear point temperatures were then extrapolated to the zero heating rate in order to estimate equilibrium solubility values.

### Primary Nucleation Induction Times

Following determination of the metastable zone width, 7 values of supersaturations (*S*) were selected to determine the dependence of the induction time distribution on *S* under isothermal conditions. All experiments were carried out at 25 °C. The corresponding solubility of α-glycine used to calculate supersaturation, *S* = *C*/*C*_s_, was taken as *C*_s_ = 249.52 mg/g of water, as this was thought to be the most accurate value available in the literature for this system.^18^ Induction time measurements were performed using the Crystalline (Technobis Crystallization Systems) instrument at a 3 mL volume. Between 18 and 25 induction time experiments were performed at each supersaturation. A series of stock solutions of glycine in deionized water were prepared directly in Crystalline vials at glycine concentrations, calculated in order to reach the desired values of supersaturation at 25 °C. The vials were then placed into the instrument, and the solid material was dispersed by a magnetic stirrer at 700 rpm and dissolved at 55 °C for a period of 30 min. Complete dissolution was confirmed by the transmissivity value reaching 100%. The temperature was then reduced to 25 °C at a cooling rate of 5 °C/min. Once the temperature reached the desired value of 25 °C, vials were held under isothermal conditions for a period of 4 h with stirring continued throughout. The isothermal induction time was recorded as a time elapsed from the start of the holding period (designated as *t*_0_) until the transmissivity value decreased below 50%. The Crystalline instrument’s built-in camera was checked to ensure that the measured transmissivity was representative of the state of recrystallization in the vials. This procedure was cycled numerous times due to the stochasticity of primary nucleation.

### Estimation of Primary Nucleation Rates

Due to the stochastic nature of primary nucleation events, a statistical distribution can be expected for the measured induction times under given conditions.^1,2^ When determining primary nucleation rates from isothermal induction time measurements, it is usually assumed that there is a constant primary nucleation rate *J*, over the induction time measurement period and that it does not vary significantly among the different vials under identical conditions. As long as the characteristic time of nucleation 1/*JV*, where *V* is the solution volume, is comparable to experimental timescales used for induction time measurements, it can be expected that there is typically a single primary nucleation event in each nucleated vial, followed by a delay, which corresponds to the time required for the nucleus to grow to the size where the resulting crystal becomes observable. Under typical agitation conditions, the nucleated crystal become observable indirectly, when it grows sufficiently large to induce sufficiently fast secondary nucleation, resulting in the formation of many small crystals, which is recorded as the induction time. Assuming that there is a single primary nucleation event in each nucleated vial, there is a delay between the primary nucleation event and the induction time detection (designated as growth time *t*_g_), which is the same in each nucleated vial; the cumulative probability of induction times *P*(*t*) follows the exponential distribution and can be described through the following equation^19^
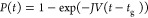
1The probability distribution *P*(*t*) can be estimated from repeated induction time experiments under same conditions, as expressed through eq 2
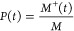
2where *M* is the total number of experiments, while *M*^+^ is the number of experiments in which nucleation was detected at the time less of equal *t*. eq 1 was fitted to the measured distribution of induction times through least-squares regression using the Levenberg–Marquardt algorithm (OriginPro 2022b). The solution volume *V* was 3 mL, while both the primary nucleation rate and the growth time were taken as fitting parameters.

### Seed Crystal Growth and Characterization

Following completion of the solubility and metastable zone width measurements, the vials were removed from the Crystal 16 instrument and placed directly onto the bench top located in a fume hood maintained at 21 °C for crystal growth to occur without agitation.^20^ Once single crystals were observed, they were removed from the vial and added to a supersaturated solution for further growth in order to reach the desired seed crystal size. The typical seed crystal size produced was 2.5 mm with the maximum seed-to-seed variations of ±1.0 mm. Single crystals were removed from the vials using a spatula placed onto a plastic Petri dish and allowed to dry in open air. Dry crystals were analyzed with a Leica DM6000M 1 optical microscope, to confirm their morphological features such as a typical bipyramidal shape, the crystal size, and that a single crystal rather than an agglomerate is present. This was followed by Raman analysis using a RXN1 Raman Spectrometer and PhAT probe (Kaiser Optical Systems) on randomly selected seed crystals. Comparison with literature Raman spectra^21^ showed that all seed crystals were the α-glycine polymorph, as expected under agitated crystallization conditions in this system.^20^

### Single-Crystal Seeding

For each seeded sample, a single-crystal seed was added to a vial containing 3 mL of solution at the glycine concentration required to obtain the desired supersaturation at the experimental temperature of 25 °C. Before addition of the seed crystal to the vial, a syringe was used to wash the seed crystal with pure water to avoid initial breeding taking place. The vials were agitated at 700 rpm throughout, and the seed was added to an agitated solution at the start of the isothermal holding period (time *t*_0_) once the temperature reached 25 °C. From a single seeded experiment, it is possible to rapidly determine both crystal growth and secondary nucleation kinetics (see below), even under conditions where primary nucleation would be too slow to measure and would not initiate crystallization at convenient timescales. The in situ camera allows real-time monitoring of the progress of the experiment. An example set of images are displayed in Supporting Information (SI), showing the sample before and immediately after seed addition, and once secondary nucleation has taken place. The Crystalline instrument’s image analysis algorithm enables data to be saved as both a number-weighted particle size distribution and a particle count against time. From these two data sets, it is possible to process the data in order to estimate both crystal growth and secondary nucleation rates.

### Secondary Nucleation Kinetics

The secondary nucleation rate, defined by the rate of increase in the number of crystals in a given volume, was measured over a range of experimental supersaturations by preparing 3 or more individual samples at each experimental supersaturation, adding a single seed crystal to each sample and measuring the increase in the number of visible crystals over time. From the primary nucleation induction times measured under the same conditions (see above), we can select conditions to ensure that no primary nucleation takes place during the secondary nucleation measurements. Experiments were conducted in 3 mL volumes under agitation by a magnetic stirrer using the Crystalline instrument, obtaining the number of crystals in the observed volume from analysis of images taken with the instrument’s built-in camera. All measurements were obtained under isothermal conditions at 25 °C, i.e., matching the conditions of the induction time measurements.

The solution, at a given concentration to provide the required supersaturation at 25 °C, was first heated to 55 °C and held for a period of 30 min to ensure full dissolution. The temperature was then reduced to 25 °C at a rate of 5 °C/min to reach the desired supersaturation. At this point, a single-crystal seed of a known size (2.5 ± 1 mm) was added to each vial while the solution was constantly agitated. This size was chosen based on a balance between ease of manipulation without damaging the seed and the convenience of being able to fit the seed into the experimental vials. The Crystalline instrument’s image analysis software provides a particle count as a function of time. Particle counts below 10 were subject to background effects, while the maximum number of particles in focus to obtain an accurate particle count using the Crystalline imaging software was found to be about 160–180. The crystal seed is subject to growth once placed in the supersaturated solution, and it was calculated that the change in solution concentration was less than 1% of the overall solution concentration within the duration of data collection for secondary nucleation and crystal growth rate experiments under conditions used here. A calibration curve was previously determined for estimation of the particle number density in the vial from particle count data.^7^ The time window to be used was selected by consideration of the limits of the image analysis, and the secondary nucleation rate was determined over a period of time where the particle count increased from 10 to 160. The secondary nucleation rate (*B*) was calculated from the rate of change of the particle number density in time.^7^

### Crystal Growth Rates

The growth rates of α-glycine crystals as a function of supersaturation were also determined from image analysis of the camera output of the Crystalline instrument,^7^ which was monitoring the population of new crystals formed by secondary nucleation, while the original seed crystal would not be normally captured by the camera. The image analysis software determines visible particle sizes, and for each time-stamped image, it produces a number-based size distribution histogram in 99 bins, linearly spanning the size range from 3 to 300 μm. Seeded experiments were completed at a range of experimental supersaturations. A MATLAB script was used to convert the number-weighted particle size distribution data into volume weighted *d*_90_ values. The *d*_90_ value gives the size where 90% (by volume) of the particles are smaller than this value. Following the onset of nucleation and above 10 particles present in the image, the change in *d*_90_ was then plotted against time until the maximum number of 160 particles in the image, and the growth rate was estimated from the change in size over time given by the slope. While the overall particle size distribution is also affected by nucleation, we are using *d*_90_ to effectively monitor the size of the largest particles present in the system, which should not be directly influenced by nucleation. By following the seeding procedure previously outlined, it is possible to estimate both secondary nucleation and growth kinetics from the same experiment in a seeded vial. Significantly, the approach used in this study enables measurement of both crystal growth and secondary nucleation rates from the same experiment even in unseeded vials, where the initial crystal is produced via primary nucleation.

### Crystallization Kinetics Assessment Workflow

**Figure 1:**
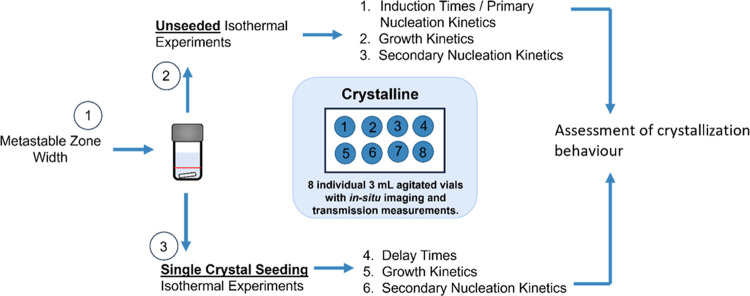
Overview of the workflow used to rapidly assess the crystallization behavior through estimation of primary and secondary nucleation and growth kinetics at given crystallization conditions.

The schematic outlined in Figure 1 provides an overview of the experimental workflow proposed in this work to rapidly screen and quantify primary and secondary nucleation and crystal growth kinetics. This approach illustrates how a few small vial experiments could be used as a starting point in crystallization process development and provide initial estimates of nucleation and crystal growth kinetics offering useful insights into crystallization behavior and aiding crystallization process design.

## Results and Discussion

### Solubility and Metastable Zone Width

**Figure 2:**
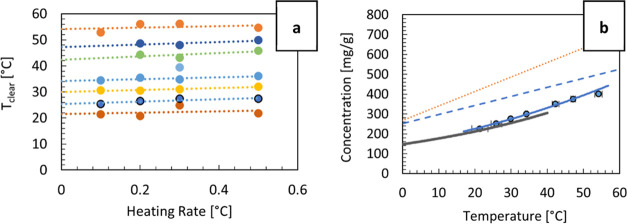
(a) Clear point temperature dependence on heating rate for glycine solutions with concentrations between 225 (red circles), 250 (dark blue circles with black border), 275 (yellow circles), 300 (light blue circles), 350 (green circles), 375 (dark blue circles), and 400 mg/g (orange circles). Solubility temperatures were estimated by extrapolating to the zero heating rate using linear regression, as indicated by dotted lines. (b) Estimated solubility temperatures and metastable zones of α-glycine in water. Temperature dependence of α-glycine solubility in water based on a thermodynamic model from Rowland^18^ is shown as a solid black line, and the van’t Hoff equation has been used for fitting the experimental data (solid blue line). Approximate metastable zone boundaries in agitated 1 mL vials at cooling rates of 0.1 and 0.5 °C/min are shown as dashed and dotted lines, respectively. Error bars are displayed in terms of confidence intervals from the solubility temperature estimations.

Solubility of α-glycine in water was estimated from clear point temperatures measured at several different heating rates between 0.1 and 0.5 °C/min over a wide range of glycine concentrations. For concentrations up to about 400 mg/g of water, there was good reproducibility from three repetitions (±0.5 °C) of clear point temperatures over repeated experiments, and dependence of clear point temperatures on the heating rate is shown in Figure 2a. Dissolution of suspended crystals during heating is kinetically limited, usually through mass and/or heat transport limitations, and therefore it is desirable to check the dependence of clear point temperatures on the heating rate and extrapolate to the zero heating rate in order to get an estimate of the solubility temperature corresponding to the thermodynamic equilibrium.

Estimated solubilities of α-glycine in water for concentrations up to 400 mg/g are shown in Figure 2b. They are in good agreement with previously reported values for solubility of α-glycine in water.^22^ Also shown is a temperature dependence of α-glycine solubility based on a thermodynamic model from Rowland, which is thought to be the most accurate value available in the literature for this system,^18^ albeit only applicable for temperatures up to 40 °C. It can be seen that the solubilities estimated from clear point measurements in this work tend to be slightly higher than those from Rowland, which may be caused by the limited sensitivity of the transmission-based method to very small amounts of suspended solids shortly before the equilibrium solubility temperature is reached.^23^

The difference between the solubility temperature and the cloud point temperature can be taken as an indication of the metastable zone width. It can be seen in Figure 2b that the metastable zone is relatively wide at this scale, corresponding to about 20 °C for the cooling rate of 0.1 °C/min and 35 °C for the cooling rate of 0.5 °C/min. This is somewhat wider than what has been previously reported in the literature for the 1 L scale (about 10 and 20 °C for the cooling rate of 0.1 and 0.5 °C/min, respectively).^24^ However, the solution volume used here was 1000 times smaller than that used in the previous literature, and thus slower nucleation and wider metastable zone would be expected, assuming that the overall primary nucleation rate is proportional to solution volume. The metastable zone is expected to be wider still for the much higher cooling rate of 5 °C/min which was used for primary and secondary nucleation studies in this work, although all concentrations investigated are well within the metastable zone for the slowest cooling rate of 0.1 °C/min. The complete data set for clear and cloud point temperatures is shown in SI, where it can be seen that cloud point temperatures from repeated experiments are widely distributed, which indicates a significant stochastic element due to primary nucleation under cooling rates deployed here.

### Induction Times and Primary Nucleation Kinetics

**Figure 3:**
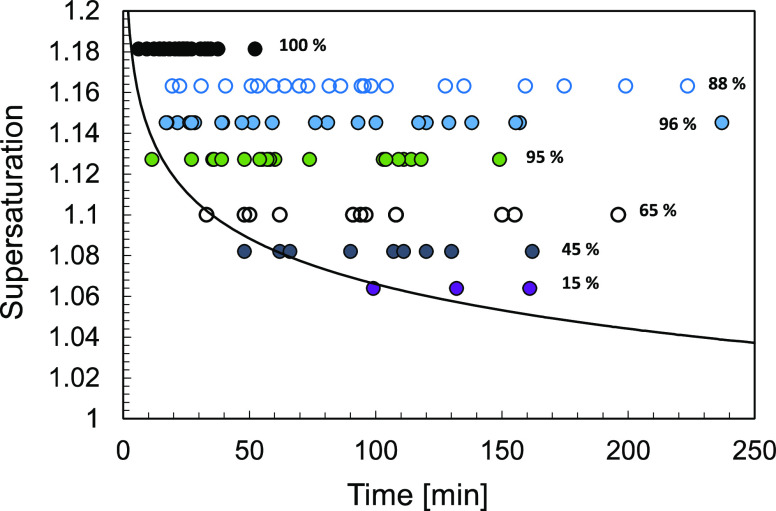
Induction times recorded at 7 different supersaturations (S) between 1.06 and 1.18 for α-glycine in water at 25 °C. Between 18 and 25 samples were measured per each supersaturation in 3 mL vials. Percentages show the proportion of the vials where nucleation was observed within 4 h. Solid line shows a theoretical growth time it takes to reach the estimated minimum crystal size to initiate secondary nucleation (see Figure 5) using the estimated growth rate as a function of supersaturation using the power law fit from Figure 4 (see text for further details).

Induction time measurements provide information on the likelihood of primary nucleation in the metastable zone under isothermal conditions. Figure 3 shows induction times recorded at each supersaturation (*S*), together with the percentage of vials that have nucleated within a 4 h time frame. At the highest *S* of 1.18, all of the vials nucleated within 60 min. However, at the lowest *S* of 1.06, the earliest vial nucleated after about 100 min, and only 15% of the vials nucleated within 4 h.

**Figure 4:**
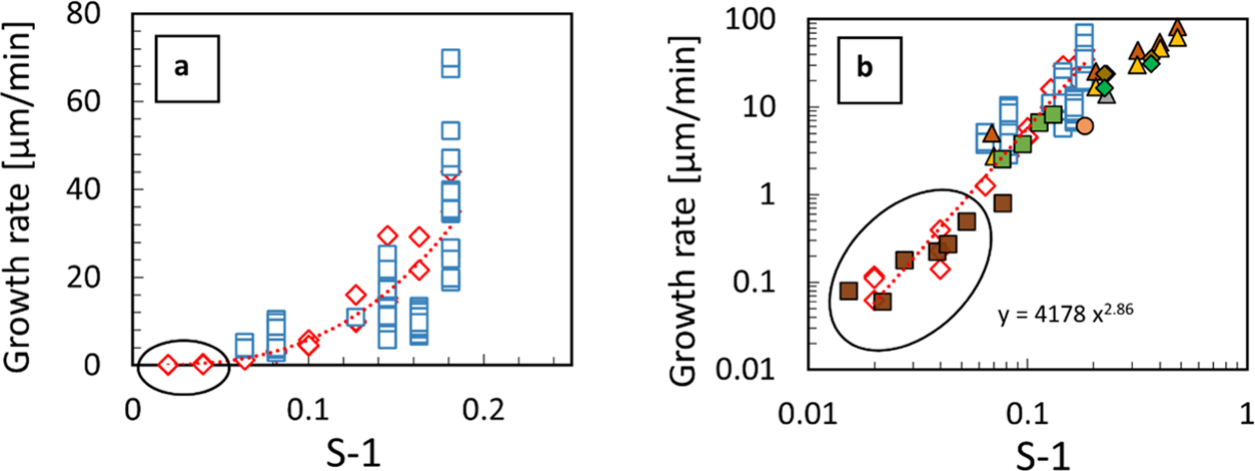
Overall crystal growth rates for α-glycine in water vs supersaturation (in terms of S*-*1) at 25 °C. (a) Measurements from seeded (open red diamonds) and unseeded (open blue squares) experiments at 9 values of supersaturations *S* ranging from 1.02 to 1.18. On a linear scale, it appears that there is a growth rate threshold below around *S* = 1.05, indicated by a black oval. (b) Same results from seeded (open red diamonds) and unseeded experiments (open blue squares) are compared with values taken from the literature using various methods^21,28−31^ with growth rate and supersaturation now plotted on a log scale, demonstrating a power law dependence across the whole range of supersaturations investigated rather than a growth rate threshold or dead zone.

**Figure 5:**
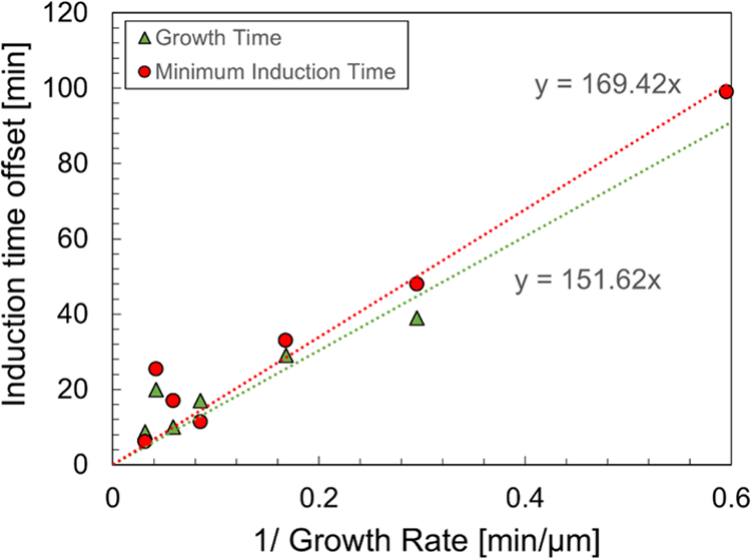
Relationship between the induction time offset for unseeded experiments and the inverse of the estimated growth rate (using the power law fit from Figure 4) at corresponding supersaturation values. The induction time offset is represented by either the minimum induction time (MIT) recorded or the growth time (*t*_g_) estimated at a given supersaturation. This allows estimation of the minimum size required to initiate secondary nucleation (169 μm based on MIT compared to 152 μm based on the growth time).

The induction times shown in Figure 3 demonstrate a typical stochastic nature of primary nucleation.^25^ It can be seen that there is a significant delay between reaching the isothermal conditions (corresponding to the time of zero in Figure 3) and the earliest induction time. This delay is indicated by a line showing a theoretical growth time *t*_g_. The line corresponds to the time required for a nucleus (following its formation at *t*_0_) to reach the minimum size to initiate secondary nucleation (estimated to be 152 μm irrespective of supersaturation, see Figure 5) applying the estimated growth rate as a function of supersaturation using the power law fit from Figure 4.

Taking the growth time offset into account, we can see that the induction times are distributed over tens to hundreds of minutes, which gives us an order of magnitude insight into the primary nucleation rate, as the *JV* term in eq 1 should then be on the order of 0.001–0.01 s per minute. In other words, the likelihood of a primary nucleation event in a given vial is on the order 0.001 to 0.01 in any given minute across the range of supersaturations considered here.

The probability distributions of induction times *P*(*t*) were fitted with the exponential distribution functional form from eq 1; see the SI for plots showing data and fits. Table 1 displays the estimated values of primary nucleation rate *J* and growth time *t*_g_ for different supersaturations.

**Table 1:** Estimated Primary Nucleation Rates *J* and Growth times *t*_g_ from the Unseeded Induction Time Experiments at 25 °C^a^

S-1	primary nucleation rate *J* [particles/(mL min)]	growth time *t*_g_ [min]
0.08	1.3 × 10^–3^ (±8.5 × 10^–5^)	37 (±4.1)
0.10	2.2 × 10^–3^ (±1.8 × 10^–4^)	29 (±4.7)
0.13	5.0 × 10^–3^ (±4.0 × 10^–4^)	17 (±2.7)
0.15	4.2 × 10^–3^ (±1.5 × 10^–4^)	10 (±1.4)
0.16	3.3 × 10^–3^ (±1.4 × 10^–4^)	20 (±1.9)
0.18	2.0 × 10^–2^ (±1.7 × 10^–3^)	8.7 (±0.81)

a The reported standard errors were obtained from the regression in OriginPro.

As expected, the primary nucleation rate *J* increases with increasing supersaturation, varying by an order of magnitude across the range of supersaturations investigated here. It can be seen that estimated primary nucleation rates do not strictly follow a monotonous trend with respect to supersaturation (the values of *J* for *S* of 1.15 and 1.16 are lower than the one for *S* of 1.13). This is most likely due to stochastic fluctuations, which are expected for nucleation processes at small volumes, especially for a relatively modest number of induction time experiments. However, even with a moderate number of induction time experiments,^26^ such as that used for the rapid assessment workflow here, there is a consistent and robust estimate of the order of magnitude of the primary nucleation rate across the range of supersaturations investigated here. It can be seen that the growth time *t*_g_ decreases with increasing supersaturation, which is also expected, as the crystal growth rate (and also the secondary nucleation rate, which contributes to rapid indirect detection of the first grown crystal at induction time) increases with supersaturation, and thus it takes a shorter time to progress from the primary nucleation event to the corresponding induction time. A similar order of magnitude (0.001–0.01 mL per minute) nucleation rates have been previously reported in literature^8,25,27^ for glycine aqueous solutions, albeit at somewhat higher supersaturations, and it should be noted that crystallization volumes, temperatures, agitation conditions, and solution preparation procedures were different in each case.

### Crystal Growth Kinetics

The overall crystal growth rates of α-glycine estimated from in situ imaging in agitated vials are presented in Figure 4a, for both unseeded and seeded experiments. The number of experimental data points varies between different conditions as only those vials which nucleated under unseeded conditions could yield crystal growth information.

Figure 4a shows estimated crystal growth rates vs supersaturation (in terms of S-1) on a linear scale. It is notable that the range of estimated growth rates from repeated experiments at a given supersaturation can be up to one order of magnitude (see Figure 4). This is not surprising in principle, as it is well known that individual crystals (of glycine and very likely other systems) can have a wide range of overall growth rates,^32^ and there are of course different growth rates for various crystal faces.^33^ As we used an ensemble-based in situ imaging method to estimate overall growth rates from relatively limited data in this work, it can be reasonably expected that the resulting estimates have somewhat limited accuracy.

Importantly, Figure 4b shows there is a good agreement, across almost three orders of magnitude from 0.1 up to 100 μm/min, between the overall crystal growth rates measured in this study and literature values that have been obtained using far more time-consuming techniques, such as in situ microscopic observations of single-crystal growth by multiple authors.^21,28−31^ It can be seen in Figure 4b that single-crystal growth rate measurements reported in the literature at the highest supersaturations appear to be somewhat lower than those obtained in this work. This may be due to increasing transport limitations at very high growth rates where agitation in our experiments enhances the mass transport from bulk solution to the surface of rapidly growing crystals compared to other experimental conditions.

It can be seen that there is a power law dependence between the overall crystal growth rate and S-1, as expected for typical crystal growth rate mechanisms,^10^ see Figure 4b, with a power law exponent of 2.86, typical of the birth and spread model.^10^ It can be also seen in Figure 4b that some other data subsets from the literature may be better described by a power law dependence with power law exponents closer to 2, but further investigation of this is beyond the scope of the present work.

In any case, a power law dependence with exponents between 2 and 3 (or higher) leads to an apparent dead zone for crystal growth, for *S* below about 1.05, when growth rate data are plotted on linear scales, as can be seen for circled data in Figure 4a. When the same data (also circled) is viewed on log scales in Figure 4b, it is clear that there is no abrupt change in the trend, but instead, it is just a feature of plotting power law dependencies in linear scales. In fact, using linear scale plots highlights the range of values measurable using a particular technique or approach. In particular, when magnitudes of experimental quantities are below the sensitivity limit of the technique, such conditions can be potentially misinterpreted as kinetic dead zones.^11^ Therefore, kinetic dead zones, cutoffs, or thresholds for either crystal growth or nucleation kinetics, when they exist in reality, cannot be reliably identified solely based on linear scale plots.

### Crystal Growth and Induction Time Offset

Induction times in unseeded isothermal experiments are statistically distributed reflecting an underlying likelihood of primary nucleation. However, the distribution of induction time is subject to an offset that corresponds to the time elapsed from the nucleation event to its detection using a particular measurement technique. This can be expressed as the growth time *t*_g_ in the cumulative probability distribution equation (eq 1), and it approximates the minimum induction time (MIT) obtained through measurements. Since for the system investigated here primary nucleation rates are relatively low (the likelihood of a primary nucleation event in a given vial is on the order of 0.001 to 0.01 in any given minute) and crystal growth rates are relatively high (on the order of 10 μm/min), it is most likely that there is only a single nucleus formed via primary nucleation in any given vial before it grows to a size where it can be detected at the corresponding induction time. In the agitated system considered here, the detection of induction time is based on extensive particle formation, which is detected as an abrupt increase in turbidity or particle counts from in situ imaging. In the case of relatively low primary nucleation rates and relatively high growth rates, this is likely to be due to secondary nucleation. This is further supported by the observation that the secondary nucleation rates per single seed crystal are much higher than primary nucleation rates in the vial for the system considered here as can be seen below, and this is also the case for other crystallization systems.^17^ Therefore, the initial single nucleus needs to grow to a crystal of sufficient size to initiate secondary nucleation at a sufficient rate to be detected, and the time it takes to do so is responsible for the induction time offset. Of course, there are some systems where primary nucleation rates are likely to be similar to or faster than secondary nucleation rates, for example, in mixing-controlled supersaturation in antisolvent and/or reactive crystallization,^23,34,35^ where the single nucleus mechanism may not be applicable.

In Figure 5, the induction time offset, represented by either the minimum induction time (MIT) recorded or the growth time (*t*_g_) estimated from the distribution of induction times at a given supersaturation, is plotted against the inverse of the estimated growth rate at the corresponding supersaturation. Overall, there is an approximately linear relationship between the inverse of the growth rate and the induction time offset, which implies that the minimum crystal size required to initiate secondary nucleation at a sufficient rate to be detected is approximately constant across the range of supersaturations examined here.

By fitting a linear function passing through the origin (see Figure 5), it is then possible to estimate the minimum size required to initiate significant secondary nucleation, as the minimum size is the growth time times the growth rate for, and therefore the minimum size is equal to the slope of the linear function. The estimated minimum size is 169 μm based on the minimum induction time recorded compared to 152 μm based on the growth time. This indicates that the single nucleus resulting from primary nucleation needs to grow to about 150–170 μm in order to initiate secondary nucleation at a sufficient rate. This in turn implies that in crystallization processes relying on secondary nucleation for generation of new particles, seed crystals need to be of sufficient size to yield significant secondary nucleation.

### Secondary Nucleation Kinetics

**Figure 6:**
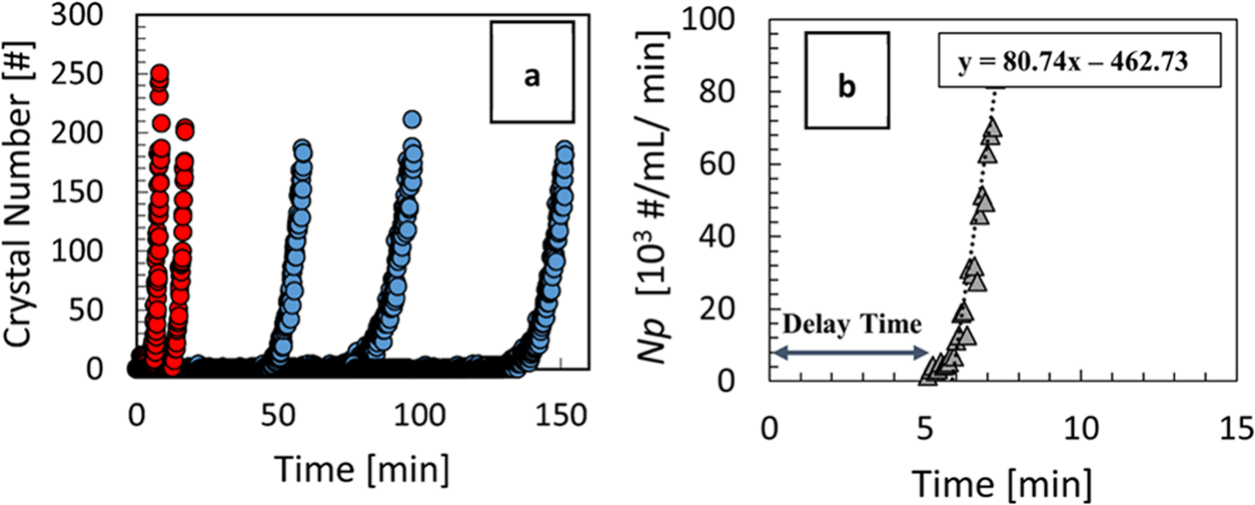
(a) Example of particle counts per image recorded for seeded (red circles) and unseeded (blue circles) experiments for *S* = 1.15. All seeded experiments resulted in rapid generation of new crystals detected after a short delay time (within 15 min), while unseeded experiments resulted in stochastic induction times. (b) Number density for one of the seeded experiments showing the delay time and the estimation of the secondary nucleation rate from the number density dependence on time.

Secondary nucleation kinetics was assessed from both seeded and unseeded experiments. Figure 6a shows an example of particle counts over time recorded by the Crystalline instrument for both seeded and unseeded experiments. While induction times for unseeded systems are widely stochastically distributed, there are much shorter delay times between the single seed crystal addition and the observed onset of extensive particle formation. It is important to clearly distinguish the induction time in unseeded experiments (see above) from the delay time in seeded experiments (see below), as they are related to different combinations of underlying physical phenomena (primary and/or secondary nucleation and crystal growth).

While the particle count in Figure 6a indicates the number of particles per image at a given time, in order to quantify secondary nucleation rate, we need to determine the corresponding particle number density in the vessel, i.e., the number of particles per unit volume. We therefore convert the particle count per image to particles per unit volume using a calibration relationship previously developed^7^ for the Crystalline instrument.

**Figure 7:**
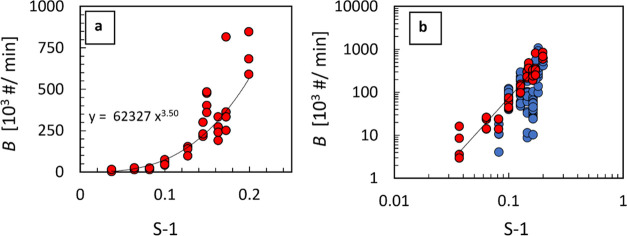
Secondary nucleation rate for α-glycine as a function of solution supersaturation expressed as S-1. (a) Secondary nucleation rate determined from seeded experiments plotted on a linear scale. (b) Secondary nucleation rate determined from both seeded and unseeded experiments plotted on a log scale. Data for seeded experiments at 10 different supersaturations between *S* = 1.04 and *S* = 1.20 (red circles) show an overall power law trend in terms of S-1. Data for unseeded experiments at 8 different supersaturations between *S* = 1.08 and *S* = 1.20 (blue circles) show significant variability, but their overall magnitude is comparable with those from seeded experiments.

Figure 6b shows how the secondary nucleation rate (*B*) is obtained from the slope of the particle count per unit volume *vs* time within a relevant time window, which is set by the limits of the particle detection and counting technique, as discussed in the secondary nucleation kinetics. Estimated secondary nucleation rates (reported per vial volume of 3 mL) are displayed as a function of supersaturation in Figure 7. We note that the secondary nucleation rates were obtained from the same experiments that provided the growth rates in Figure 4, highlighting the multitude of data that can be extracted simultaneously from rapid small-scale seeded experiments following the approach outlined here.

Secondary nucleation rates for seeded experiments show an overall power law trend in terms of S-1 with an exponent of 3.5. Similarly to the case of growth rate discussed above, a power law dependence with such an exponent results in an apparent dead zone or secondary nucleation threshold when secondary nucleation rate data are plotted on linear scales (Figure 7a). However, when the same data are plotted on logarithmic scales (Figure 7b), it is again clear that there is no abrupt change in the trend at low supersaturations, and there is no physically meaningful secondary nucleation threshold. However, there is always a limit where the secondary nucleation becomes too slow to be measurable by a given method (in this work, it is about 100 particles per mL per minute).

Figure 7b shows a comparison of secondary nucleation rates estimated from seeded and unseeded experiments at the same supersaturations. Secondary nucleation rates for seeded experiments at 10 different supersaturations between *S* = 1.04 and *S* = 1.20 show an overall power law trend in terms of S-1. Secondary nucleation rates from unseeded experiments show higher variability than those from seeded experiments at corresponding supersaturations, but the overall magnitude is comparable with those from seeded experiments.

This is perhaps unsurprising, given that (under the single nucleus mechanism framework^36,37^ which is relevant for unseeded experiments here) primary nucleation results in the formation of a single crystal followed by crystal growth until it reaches a minimum size required to initiate secondary nucleation at a sufficient rate and then acts as a seed for secondary nucleation in the “one to many” effect. There is likely to be a greater variability of crystals formed through primary nucleation under unseeded conditions compared to seed crystals externally introduced under seeded conditions, and since the secondary nucleation rate depends on the seed size,^7^ the variation of crystal sizes inducing secondary nucleation can lead to a broader distribution of unseeded secondary nucleation rates. It should be noted that this method of determining secondary nucleation kinetics is appropriate for systems in which the rate of secondary nucleation is much higher than that of primary nucleation. In the case where primary nucleation kinetics are of similar or higher magnitude than those of secondary nucleation, the rate of change of the number density of crystals will reflect both primary and secondary nucleation kinetics.

**Figure 8:**
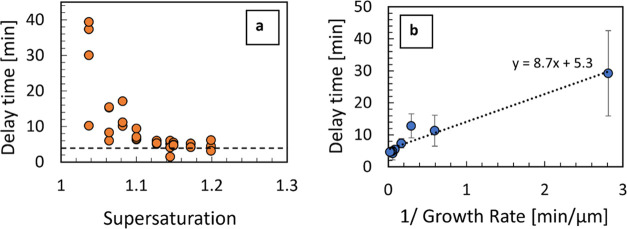
(a) Delay time for detection of secondary nucleation following single seed addition measured over a range of supersaturations. The horizontal line is to illustrate that the delay time reaches a minimum value around 4 min at higher supersaturations. (b) Secondary nucleation delay time (each point represents an average of the points in panel (a)) plotted against the inverse of the estimated growth rate calculated from the power law fit (Figure 4) for equivalent supersaturation in seeded experiments.

Figure 8a displays the supersaturation dependence of the delay time between the seed crystal addition and the time at which the particle count begins to rise steeply (see Figure 6), which is a manifestation of particle formation due to secondary nucleation. It can be seen that there is a significant variability of delay times from vial to vial, especially at lower supersaturations. Overall, the delay time decreases as supersaturation increases, but it does not seem to approach zero at higher supersaturations. Figure 8b shows the mean delay time plotted against the inverse of the estimated growth rate at corresponding supersaturations.

From the linear fit to the data in Figure 8b, it can be seen that there appears to be a minimum delay time of about 5 min, corresponding to the intercept of the linear fit at the limit of the inverse of the growth rate reaching zero. This indicates that at relatively high supersaturations, where the growth rate is high, there is a certain period required for the secondary nucleation to be initiated after the seed crystal is introduced in the supersaturated solution. This may be because the seed requires a certain time to establish a well-developed growth regime with a corresponding boundary layer in the solution adjacent to the growing crystal interface. The formation of such an interfacial solution layer may be required for secondary nucleation driven by fluid shear at the surface of a growing crystal.^38^

**Figure 9:**
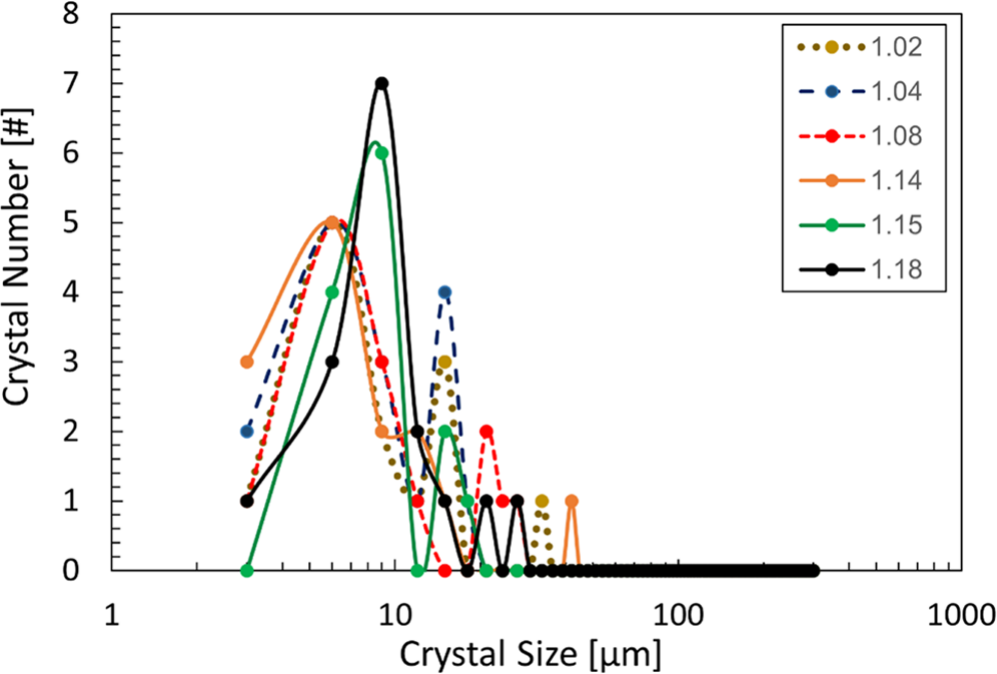
Number-weighted particle size distribution at the point of the earliest detection of secondary nucleation (>10 crystals in focus per image) in seeded experiments. Each symbol corresponds to a specific supersaturation, as described in the legend.

Furthermore, the linear fit to the data in Figure 8b suggests that there is a further delay time related to the time required for new crystals formed by secondary nucleation to grow to about 9 μm (corresponding to the slope of the linear fit). This is consistent with sizes of newly formed particles observed at the earliest detection of secondary nucleation in seeded experiments. Corresponding particle size distributions are shown in Figure 9 with principal modes of number-based particle distributions between 5 and 9 μm.

The delay time required for crystals formed by secondary nucleation to the detectable size (around 5–10 μm using the detection approach used here) should be also accounted for in the analysis of unseeded experiments, which would slightly decrease the minimum crystal size required to initiate secondary nucleation at a sufficient rate to be detected. Also, in the above analysis, we have assumed that all crystals grow at the same prevalent rate at a given supersaturation, although it is well known that there is a significant dispersion of growth rates between individual single crystals.^32^ Therefore, estimates of minimum crystal sizes above are necessarily approximations within the limits of relevant assumptions.

### Crystallization Behavior Assessment

Crystal growth and nucleation are often studied and presented as two conceptually separate processes. Our approach allows us to directly investigate relationships between the growth rate and the corresponding primary and secondary nucleation rates under same experimental conditions, based on the consistent measurement and analysis framework. The well-defined quantitative relationship between growth and nucleation kinetics is expected based on the power law dependence on supersaturation that they both tend to follow. This is also related to potential mechanistic relationships, where, for example, the secondary nucleation induced by fluid shear is related to the solution boundary layer at the surface of a growing crystal in contact with the supersaturated solution, where loosely bound clusters are swept from the boundary layer and serve as crystal nuclei.

**Figure 10:**
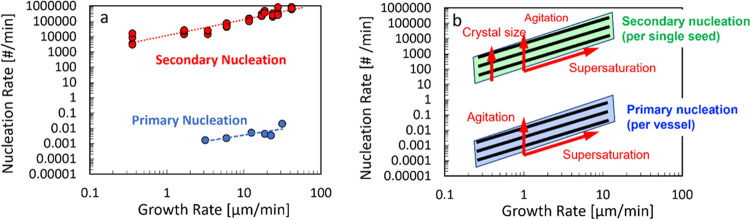
(a) Assessment of crystallization behavior of α-glycine based on nucleation and growth rates at corresponding crystallization conditions in magnetically agitated vials. Each point represents both the nucleation and estimated growth rate at the same supersaturation. Seeded secondary nucleation rates (per single seed crystal in a 3 mL vial) are shown in red, and unseeded primary nucleation rates (per 3 mL vial) are shown in blue. (b) Schematic of crystallization behavior assessment showing expected dependencies of nucleation and growth rates on the supersaturation, agitation, and seed crystal size represented by respective arrows indicating the direction of increasing magnitude.

For the case of crystallization of α-glycine from aqueous solutions in magnetically agitated vials, as shown in Figure 10, the secondary nucleation rates are up to 6 orders of magnitude higher than the primary nucleation rates. It is important to note that in systems where secondary nucleation rates are much higher than primary nucleation rates,^17^ the single nucleus mechanism is expected to be an appropriate conceptual framework for interpretation of nucleation behavior and analysis of nucleation experiments.

A general assessment of crystallization behavior can be presented in terms of the nucleation rate dependence on the corresponding growth rate, as shown in Figure 10a. In this diagram, every point corresponds to nucleation and crystal growth rates determined at a particular supersaturation under given crystallization conditions. When using any particular method to estimate magnitudes of nucleation and growth rates, it is important to keep in mind what the range of values that can be measured using a given approach. For example, when primary nucleation induction times can be measured over certain periods of time, only a certain range of *JV* magnitudes is accessible. If induction times can be measured between minutes and hundreds of minutes, then the corresponding *JV* values are on the order 0.001 to 0.1 per minute. That implies that neither slower nor faster primary nucleation rates would not be practically measurable by this approach. Similarly, when secondary nucleation rates are assessed based on a certain range of the number of particles in an image over times ranging from minutes to seconds to hundreds of seconds, only a certain range of *B* magnitudes is accessible. It is important to consider what effects processing conditions have on primary and secondary nucleation and crystal growth kinetics, as illustrated in Figure 10b. First of all, as supersaturation increases, both crystal growth and nucleation rates increase in tandem, regardless of the system, as indicated by the corresponding arrow in Figure 10b. Furthermore, as the agitation rate increases, the primary nucleation rate increases significantly for this system,^8,9,26^ and the secondary nucleation rate is expected to increase as well,^39^ while the growth rate may also increase somewhat due to enhanced convective mass transfer. This would be expected to move the dependence of both the primary and secondary nucleation rates on the growth rate upward, as indicated in Figure 10b. Also, it is worth considering the effect of seed crystal size, where the secondary nucleation rate increases with crystal size,^7^ while the effect of crystal size on the growth rate is expected to be much less significant. This would then be expected to move the dependence of the secondary nucleation rate on the growth rate upward, as indicated in Figure 10b. These relationships can be explored to rationally manipulate crystallization conditions for achieving desirable outcomes in batch or continuous crystallization processes.

As previously proposed,^15,16^ it is possible to classify crystallization behaviors into four categories in relation to their growth (slow or fast) and nucleation (slow or fast) kinetics. These categories can be based on a chosen characteristic time (τ), which may be based on a relevant process residence time and a characteristic length (*L*), which may be based on a target crystal size, where the crossover nucleation and growth rates would be 1/τ and *L*/τ, respectively.

While we illustrated the workflow for rapid assessment of nucleation and growth kinetics for small-scale magnetically agitated vials, a similar approach can be used across multiple scales, where estimates of primary and secondary nucleation and growth rates can be obtained as well. For example, primary nucleation rates were investigated across scales recently in both sub mL quiescent systems^25^ and agitated systems up to 10 L,^40^ where it can be seen that either *J* or *JV* values have similar orders of magnitude across a wide range of scales.

More generally, in order to identify conditions suitable for particular crystallization processes, certain combinations of crystal nucleation and growth rates are required to provide desirable crystallization outcomes, such as crystal size and yield, at reasonable residence times.^41^ For example, it is instructive to consider what orders of magnitude are required for nucleation rates at the steady-state operation of a single-stage continuous stirred tank (or mixed suspension mixed product removal) crystallizer. Relationships between the steady-state solid volume fraction ϕ, the (volume weighted) mean crystal size *d*_4,3_ and the corresponding crystal growth rate *G* and the (overall) nucleation rate *B* at the steady-state supersaturation and the mean residence time τ can be described for cubic crystals as follows^41^
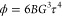

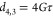
From this, we can express the desired values of *B* and *G* in terms of ϕ, *d*_4,3_, and τ as follows
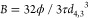

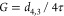
Taking some typical values, ϕ = 0.1, *d*_4,3_ = 10^–4^ m, τ = 1000 s, we get *B* = 6.4 × 10^4^ /(mL min) and *G* = 1.5 μm/min. From the data shown in Figure 10, we can see that such a combination of nucleation and growth rates, in terms of orders of magnitude, may indeed be accessible for the system investigated here, when secondary nucleation is considered. The required supersaturation *S*, based on the desired value of *G*, would be around 1.06 (based on the power law fit from Figure 4b), so the corresponding value of *B* for a single seed crystal would be around 10^3^ to 10^4^/(mL min) based on data shown in Figure 7b. Based on the rapid kinetic assessment of this system, it would therefore be plausible to guide the process development for a single-stage stirred tank crystallizer to operate around *S* = 1.06 and assess the secondary nucleation at a suitable scale around this supersaturation. We note that the primary nucleation kinetics at these conditions is likely to be extremely slow, around 10^–3^ to 10^–4^ /(mL min), so it would not be practical to rely on unseeded experiments in small volumes to estimate relevant crystallization kinetics data.

## Conclusions

This study has outlined the development and validation of an experimental workflow enabling the rapid assessment of primary and secondary nucleation and growth kinetics, using an example of cooling crystallization of α-glycine from aqueous solutions in magnetically agitated vials.

First, an assessment of primary nucleation kinetics based on unseeded experiments was conducted, and suitable conditions for seeded experiments were determined. Single-crystal seeded experiments were then used to assess secondary nucleation kinetics. An image analysis algorithm provided particle counts and size distributions for both seeded and unseeded experiments and allowed quantification of secondary nucleation and crystal growth rates. Primary nucleation rates were determined from distributions of induction times obtained from unseeded experiments. Interdependencies of primary and secondary nucleation and crystal growth kinetics were carefully considered and quantitatively analyzed to clearly distinguish and decouple their respective kinetics.

It was found that secondary nucleation rates were several orders of magnitude higher than primary nucleation rates in the magnetically stirred vials used here, and therefore the single nucleus mechanism is appropriate to interpret and analyze nucleation behavior in this system. Crystal growth rates estimated from in situ image analysis were comparable with data from previous literature based on single-crystal growth measurements across three orders of magnitude. Both growth rates and nucleation rates show power law dependencies on supersaturation and neither indicates any dead zones or thresholds which misleadingly appear to be present when data is plotted on linear scales. This highlights the need to use logarithmic scale plots when analyzing such data and consider sensitivity (what is the minimum value measurable using a given approach) and limitations of any particular method used to determine nucleation or growth rates.

Here, we illustrated the workflow for rapid assessment of nucleation and growth kinetics for small-scale magnetically agitated vials, which can also be used to quantify the effects of agitation and seed crystal size on crystallization kinetics. A similar approach can be deployed across multiple scales, where estimates of primary and secondary nucleation and growth rates can be obtained as well, in order to facilitate scale-up of crystallization processes.

We used experimentally observed primary and secondary nucleation and growth rates to present a quantitative assessment of crystallization behavior, which revealed clearly defined quantitative relationships between nucleation and growth kinetics determined at given crystallization conditions. These relationships are based on power law dependences on supersaturation that both growth and nucleation kinetics tend to follow. Such relationships therefore provide useful insights into the crystallization behavior. They can also be explored to rationally manipulate crystallization conditions for achieving desirable outcomes in crystallization processes, as certain combinations of crystal nucleation and growth rates are required to provide desirable crystallization outcomes, such as crystal size and yield, at reasonable residence times.

## Supporting Information Available

Transmission trace for 4 different concentration solutions (Figure S1); characteristic IR spectra for α (red) and γ-glycine (blue) (Figure S2); characteristic powder X-ray diffraction pattern for (a) for α-glycine and (b) γ-glycine (Figure S3); the extrapolated solubility values recorded from the Crystal 16 (Table S1); the metastable zone width is shown at 4 different cooling rates ranging from 0.1–0.5°C/ min (Figure S4); cumulative probability distributions of the stochastic induction time data for the glycine in water system across multiple supersaturations (Figure S5); the primary nucleation rate J and growth time *t*_g_ (Figure S6); a schematic of a typical temperature seeding profile (Figure S8); example Raman spectra for α-glycine with characteristic peaks (Figure S9); images using the Crystalline instrument, 3 mL vial sample at *S* = 1.17 showing a single-seeded secondary nucleation experiment (Figure S10); the secondary nucleation rate for α-glycine as a function of solution supersaturation expressed as S-1 (Figure S11) (PDF)

## References

[ref1] Devos C.; Van Gerven T.; Kuhn S. A Review of Experimental Methods for Nucleation Rate Determination in Large-Volume Batch and Micro fluidic Crystallization. Cryst Growth Des. 2021, 21, 2541–2565. 10.1021/acs.cgd.0c01606.

[ref2] Kadam S. S.; Kulkarni S. A.; Ribera R. C.; Stankiewicz A. I.; ter Horst J. H.; Kramer H. J. M. A new view on the metastable zone width during cooling crystallization. Chem. Eng. Sci. 2012, 72, 10–19. 10.1016/j.ces.2012.01.002.

[ref3] Sangwal K. Novel approach to analyze metastable zone width determined by the polythermal method: physical interpretation of various parameters. Cryst. Growth Des. 2009, 9, 942–950. 10.1021/cg800704y.

[ref4] Mitchell N. A.; Frawley P. J. Nucleation kinetics of paracetamol ethanol solutions from metastable zone widths. J. Cryst. Growth 2010, 312, 2740–2746. 10.1016/j.jcrysgro.2010.05.043.

[ref5] Brandel C.; ter Horst J. H. Measuring induction times and crystal nucleation rates. Faraday Discuss. 2015, 179, 199–214. 10.1039/C4FD00230J. 25865429

[ref6] Cedeno R.; Maosoongnern S.; Flood A. Direct Measurements of Primary Nucleation Rates of p-Aminobenzoic Acid and Glutamic Acid and Comparison with Predictions from Induction Time Distributions. Ind. Eng. Chem. Res. 2018, 57, 17504–17515. 10.1021/acs.iecr.8b03625.

[ref7] Briuglia M. L.; Sefcik J.; ter Horst J. H. Measuring secondary nucleation through single crystal seeding. Cryst. Growth Des. 2019, 19, 421–429. 10.1021/acs.cgd.8b01515.

[ref8] Forsyth C.; Burns I. S.; Mulheran P. A.; Sefcik J. Scaling of Glycine Nucleation Kinetics with Shear Rate and Glass-Liquid Interfacial Area. Cryst. Growth Des. 2016, 16, 136–144. 10.1021/acs.cgd.5b01042.

[ref9] Sheridan R.; Cardona J.; Tachtatzis C.; Chen Y. C.; Cleary A.; Briggs N.; et al. Effect of oscillatory flow conditions on crystalliser fouling investigated through non-invasive imaging. Chem. Eng. Sci. 2022, 252, 117188 10.1016/j.ces.2021.117188.

[ref10] Mullin J. W. Crystallisation, 4th Ed. Butterworth Heinemann: Oxford, UK, 2001.

[ref11] Liu Y.; Black J. F. B.; Boon K. F.; Cruz-Cabeza A. J.; Davey R. J.; Dowling R. J.; et al. When Crystals Do Not Grow: The Growth Dead Zone. Cryst. Growth Des. 2019, 19, 4579–4587. 10.1021/acs.cgd.9b00478.

[ref12] Srisa-Nga S.; Flood A. E.; White E. T. The secondary nucleation threshold and crystal growth of α-glucose monohydrate in aqueous solution. Cryst. Growth Des. 2006, 6, 795–801. 10.1021/cg050432r.

[ref13] Brown C. J.; Mcglone T.; Yerdelen S.; Srirambhatla V.; Mabbott F.; Gurung R.; et al. Enabling precision manufacturing of active pharmaceutical ingredients: workflow for seeded cooling continuous crystallisations. Mol. Syst. Des. Eng. 2018, 3, 421–592. 10.1039/C7ME00096K.

[ref14] Arruda R. J.; Cally P. A. J.; Wylie A.; Shah N.; Joel I.; Leff Z. A.; et al. Automated and Material-Sparing Workflow for the Measurement of Crystal Nucleation and Growth Kinetics. Cryst. Growth Des. 2023, 23, 3845–3861. 10.1021/acs.cgd.3c00252.

[ref15] Acevedo D.; Nagy Z. K. Systematic classification of unseeded batch crystallization systems for achievable shape and size analysis. J. Cryst. Growth 2014, 394, 97–105. 10.1016/j.jcrysgro.2014.02.024.

[ref16] Rathi S.; Chavan R. B.; Shastri N. R. Classification of the crystallization tendency of active pharmaceutical ingredients (APIs) and nutraceuticals based on their nucleation and crystal growth behaviour in solution state. Drug Delivery Transl. Res. 2020, 10, 70–82. 10.1007/s13346-019-00663-w. 31407270

[ref17] Hoffmann J.; Flannigan J.; Cashmore A.; Briuglia M. L.; Steendam R. R. E.; Gerard C. J. J.; et al. The unexpected dominance of secondary over primary nucleation. Faraday Discuss. 2022, 235, 2166–2179. 10.1039/D1FD00098E. 35388815

[ref18] Rowland D. Thermodynamic Properties of the Glycine + H2O System. J. Phys. Chem. Ref. Data 2018, 47, 023104 10.1063/1.5016677.

[ref19] Jiang S.; ter Horst J. H. Crystal nucleation rates from probability distributions of induction times. Cryst. Growth Des. 2011, 11, 256–261. 10.1021/cg101213q.

[ref20] Vesga M. J.; McKechnie D.; Mulheran P. A.; Johnston K.; Sefcik J. Conundrum of γ glycine nucleation revisited: to stir or not to stir?. CrystEngComm 2019, 21, 2234–2243. 10.1039/C8CE01829D.

[ref21] Shiau L. D. Determination of the nucleation and growth kinetics for aqueous L-glycine solutions from the turbidity induction time data. Crystals 2018, 8, 403 10.3390/cryst8110403.

[ref22] Manson A.; Sefcik J.; Lue L. Temperature Dependence of Solubility Predicted from Thermodynamic Data Measured at a Single Temperature: Application to α, β, and γ-Glycine. Cryst. Growth Des. 2022, 22, 1691–1706. 10.1021/acs.cgd.1c01217. PMC9008547 35431659

[ref23] Svoboda V.; Macfhionnghaile P.; Mcginty J.; Connor L. E.; Oswald I. D. H.; Sefcik J. Continuous Cocrystallization of Benzoic Acid and Isonicotinamide by Mixing-Induced Supersaturation: Exploring Opportunities between Reactive and Antisolvent Crystallization Concepts. Cryst. Growth Des. 2017, 17, 1902–1909. 10.1021/acs.cgd.6b01866.

[ref24] Bonnin-Paris J.; Bostyn S.; Havet J.-L.; Fauduet H. Determination of the Metastable Zone Width of Glycine Aqueous Solutions for Batch Crystallizations. Chem. Eng. Commun. 2011, 198, 1004–1017. 10.1080/00986445.2011.545301.

[ref25] Dela Cruz I. J. C.; Perez J. V.; Alamani B. G.; Capellades G.; Myerson A. S. Influence of Volume on the Nucleation of Model Organic Molecular Crystals through an Induction Time Approach. Cryst. Growth Des. 2021, 21, 2932–2941. 10.1021/acs.cgd.1c00101.

[ref26] Forsyth C.; Mulheran P. A.; Forsyth C.; Haw M. D.; Burns I. S.; Sefcik J. Influence of controlled fluid shear on nucleation rates in glycine aqueous solutions. Cryst. Growth Des. 2015, 15, 94–102. 10.1021/cg5008878.

[ref27] Dowling R. J. A Study of the Nucleation and Growth of glycine and DL-alanine. PhD thesis; University of Manchester, 2012.

[ref28] Dowling R.; Davey R. J.; Curtis R. A.; Han G.; Poornachary S. K.; Chow P. S.; Tan R. B. H. Acceleration of crystal growth rates: An unexpected effect of tailor-made additives. Chem. Commun. 2010, 46, 5924–5926. 10.1039/c0cc00336k. 20601977

[ref29] Li L.; Rodríguez-Hornedo N. Growth kinetics and mechanism of glycine crystals. J. Cryst. Growth 1992, 121, 33–38. 10.1016/0022-0248(92)90172-F.

[ref30] Lung-Somarriba B. L. M.; Moscosa-Santillan M.; Porte C.; Delacroix A. Effect of seeded surface area on crystal size distribution in glycine batch cooling crystallization: A seeding methodology. J. Cryst. Growth 2004, 270, 624–632. 10.1016/j.jcrysgro.2004.07.015.

[ref31] Sultana M.; Jensen K. F. Microfluidic continuous seeded crystallization: Extraction of growth kinetics and impact of impurity on morphology. Cryst. Growth Des. 2012, 12, 6260–6266. 10.1021/cg301538y.

[ref32] Little L. J.; Sear R. P.; Keddie J. L. Does the γ Polymorph of Glycine Nucleate Faster? A Quantitative Study of Nucleation from Aqueous Solution. Cryst. Growth Des. 2015, 15, 5345–5354. 10.1021/acs.cgd.5b00938.

[ref33] Offiler C. A.; Cruz-Cabeza A. J.; Davey R. J.; Vetter T. Crystal Growth Cell Incorporating Automated Image Analysis Enabling Measurement of Facet Specific Crystal Growth Rates. Cryst. Growth Des. 2022, 22, 2837–2848. 10.1021/acs.cgd.1c01019.

[ref34] McGinty J.; Chong M. W. S.; Manson A.; Brown C. J.; Nordon A.; Sefcik J. Effect of process conditions on particle size and shape in continuous antisolvent crystallisation of lovastatin. Crystals 2020, 10, 925 10.3390/cryst10100925.

[ref35] Raza S. A.; Schacht U.; Svoboda V.; Edwards D. P.; Florence A. J.; Pulham C. R.; et al. Rapid Continuous Antisolvent Crystallization of Multicomponent Systems. Cryst. Growth Des. 2018, 18, 210–218. 10.1021/acs.cgd.7b01105.

[ref36] Kadam S. S.; Kramer H. J. M.; ter Horst J. H. Combination of a single primary nucleation event and secondary nucleation in crystallization processes. Cryst. Growth Des. 2011, 11, 1271–1277. 10.1021/cg101504c.

[ref37] Kulkarni S. A.; Meekes H.; ter Horst J. H. Polymorphism control through a single nucleation event. Cryst. Growth Des. 2014, 14, 1493–1499. 10.1021/cg500059u.

[ref38] Anwar J.; Khan S.; Lindfors L. Secondary crystal nucleation: Nuclei breeding factory uncovered. Angew. Chem., Int. Ed. 2015, 54, 14681–14684. 10.1002/anie.201501216. 25809644

[ref39] Cashmore A. Understanding and Measurement of Secondary Nucleation. PhD thesis; University of Strathclyde Glasgow, 2022.

[ref40] Yerdelen S.; Yang Y.; Quon J. L.; Papageorgiou C. D.; Mitchell C.; Houson I.; et al. Machine Learning-Derived Correlations for Scale-Up and Technology Transfer of Primary Nucleation Kinetics. Cryst. Growth Des. 2023, 23, 681–693. 10.1021/acs.cgd.2c00192. PMC9896482 36747575

[ref41] Mersmann A. Crystallization Technology Handbook, 2nd ed.; CRC Press: Boca Raton, 2001; pp 145–186.

