# Supplementary material for: Rapid Assessment of Crystal Nucleation and Growth Kinetics: Comparison of Seeded and Unseeded Experiments
Source: Cryst Growth Des. 2023 Jun 20;23(7):4779–90. doi: 10.1021/acs.cgd.2c01406 (PMC10326855; doi:10.1021/acs.cgd.2c01406)
Supplement: Supplementary file 1 — cg2c01406_si_001.pdf [file cg2c01406_si_001.pdf]

# Supplementary Information:

# Rapid assessment of crystal nucleation and growth kinetics: comparison of seeded and unseeded experiments


*Andrew Cashmore[1,2], Russell Miller[1,2], Hikaru Jolliffe[2], Cameron J. Brown[2], Mei Lee[3], Mark D. Haw[1], Jan Sefcik[1,2]**

1. Department of Chemical and Process Engineering, University of Strathclyde, 75 Montrose Street, Glasgow G1 1XJ, UK
2. CMAC Future Manufacturing Research Hub, Technology and Innovation Centre, 99 George Street, Glasgow, G1 1RD, UK
3. GlaxoSmithKline, Product Development and Supply, Gunnellswood Rd, Stevenage, SG1 2NY, UK

The Crystal 16 contains 16 independent chambers with a sample capacity of 1.5 mL and enables precise and specific temperature control. Within each chamber there is a light transmission probe to detect a change in turbidity of the system which indicates formation/ dissolution of crystals. In this work, the Crystal 16 was used to measure the metastable zone width. To achieve this, temperature cycles are set, enabling the point of dissolution to be determined (solubility) on heating, and the point of recrystallisation (nucleation) on cooling (Figure 1). To determine primary nucleation rates, the Crystal 16 can be used to rapidly generate large numbers of induction time measurements. The induction time is recorded under isothermal conditions and a solution is brought to a selected temperature generating a known supersaturation. Once the supersaturation is generated, the temperature is maintained until nucleation spontaneously occurs and the time taken for this to take place is recorded.

For the rest of this work, the Crystalline system was used which, similarly to the Crystal 16, enables temperature profiles to be set however, within the Crystalline platform there is an integrated camera used alongside the transmission probe. This enables *in-line* crystal counting and size analysis. The volume is also larger than the Crystal 16 with a maximum volume of 8 mL but the productivity is halved with only 8 independent chambers.

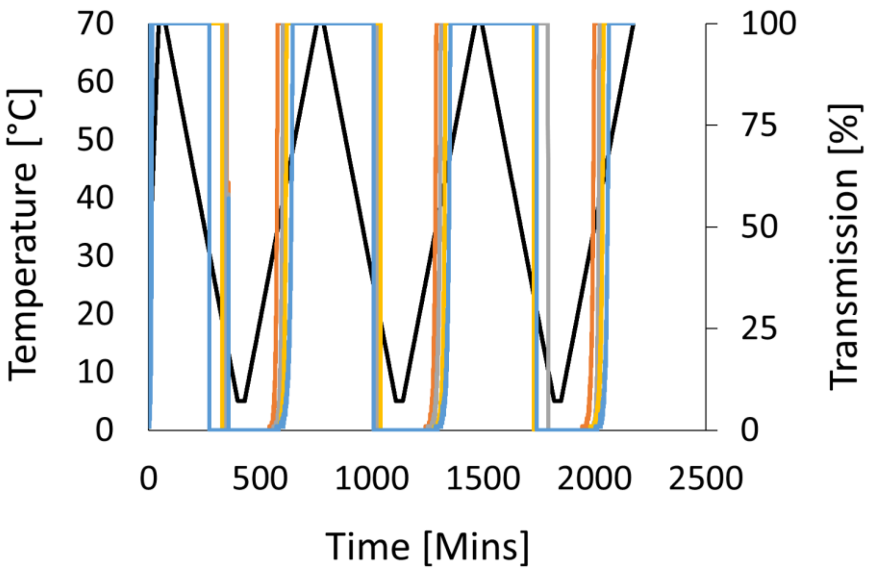


**Figure 1.** Transmission trace for 4 different concentration solutions. The tmperature is increased (black line) to 70°C to ensure complete dissolution and held for a period of 30 minutes. It is then decreased at a known cooling rate to 5°C and held for 30 minutes. It is then subsequently heated up again for dissolution and a new repeat cycle of crystallisation/ dissolution is commenced. The figure thus shows three repeat cycles each providing measurements of light transmission through each sample over the temperature cycle. The point during the temperature decrease at which transmission reduces from 100% to 0 indicates formation of crystals i.e., the temperature limit of the metastable zone for the given sample's concentration; the point during the subsequent temperature increase where transmission increases again to 100% indicates dissolution of the crystal, i.e., the temperature which the given sample's concentration equals the solubility.

The glycine used in this study was first crystallised and analysis was performed to ensure that the desired form was obtained. Characterisation was completed using a combination of Attenuated Total Reflectance Fourier Transform Infrared Spectroscopy (ATR FTIR) and powder X-ray diffraction (PXRD) and for both of these techniques the crystals were firstly ground into a fine powder. All spectra were recorded under ambient conditions.

Characteristic IR peaks were identified for the α polymorphic form at 907 $cm^{-1}$, with γ showing a characteristic peak at 927 $cm^{-1}$. There was also a shared peak at 887 $cm^{-1}$ as shown below in the example spectra (Figure 2).

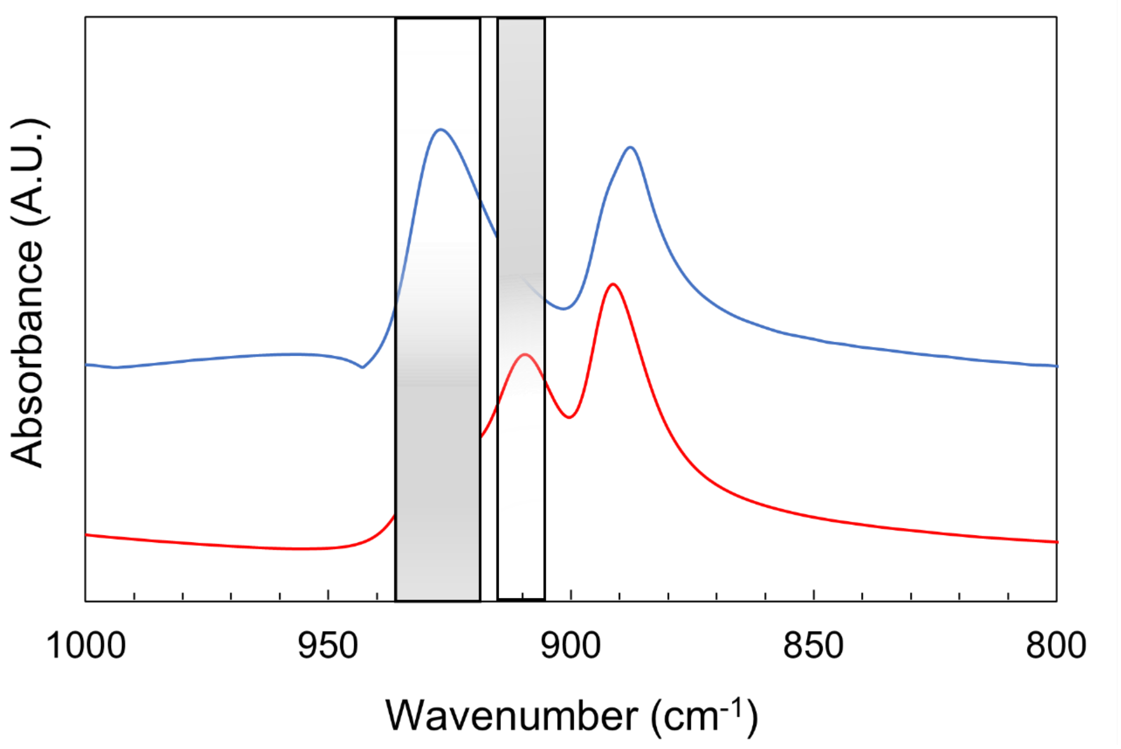


**Figure 2**. Characteristic IR spectra for α (red) and γ-glycine (blue)**.** The grey shaded boxes represent the characteristic peaks. Peaks are visible at 910 $cm^{-1}$ for α-glycine and 928 $cm^{-1}$ γ-glycine.

The PXRD pattern (Figure 3) highlighted peaks at 2θ of 19° and 30° in correlation with what has been seen in literature for α-glycine[9] and 22° and 25.5° for γ glycine. The characteristic diffraction is shown in Figure 3.2. confirming that the correct form can be selectively obtained following the crystallisation methods described in this work.

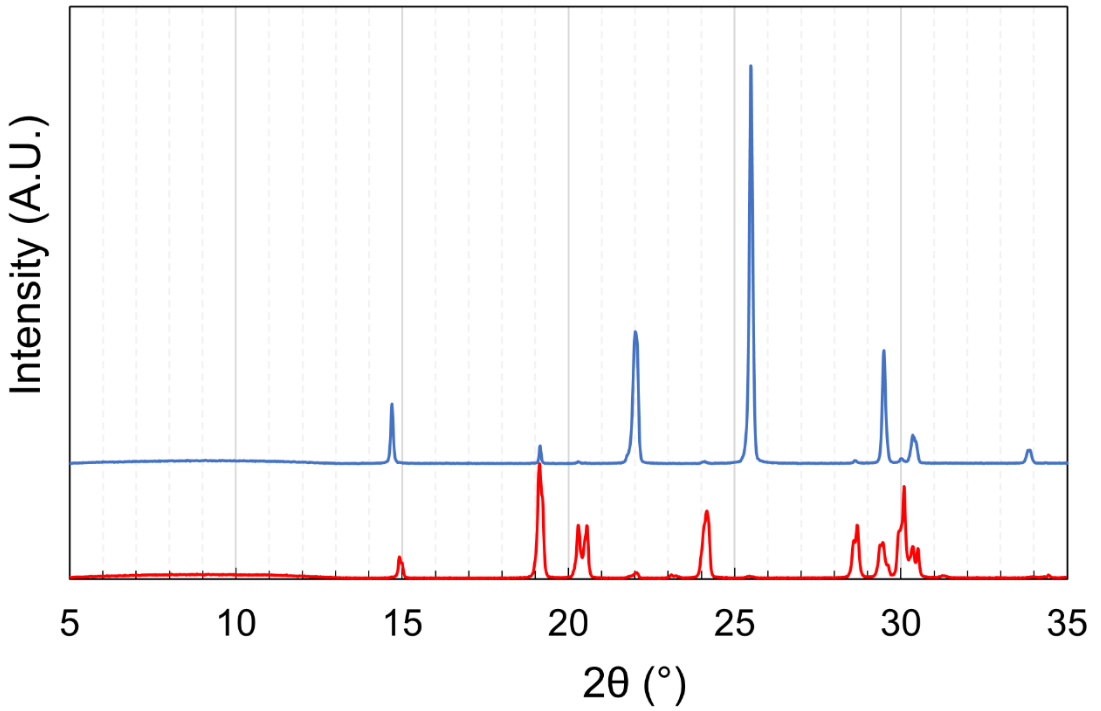


**Figure 3.** *C*haracteristic powder X-ray diffraction pattern for (a) for α-glycine and (b) γ-glycine. Characteristic peaks at 2θ of 19 and 30 α-glycine and 21° and 25.3° for γ glycine.

In order to estimate the ‘true equilibrium solubility’, the polythermal technique was performed at multiple heating rates and an extrapolation performed to a theoretical ‘0°C/ min’ heating rate. The ‘true’ equilibrium solubility values recorded in this work are displayed in Table 1. with the corresponding concentration and the confidence interval on the intercept. It is clear that it is necessary to take the heating rate into account when conducting polythermal measurements of solubility.

**Table 1.** The extrapolated solubility values recorded from the Crystal 16. The solubility column here is taken from the intercept and the standard error is shown in relation to this value.

| **Concentration (mg/g)** | **Solubility Temperature (°C)** | **Standard Error (±)** |
|---|---|---|
| 225 | 21.38 | 2.24 |
| 250 | 25.94 | 1.48 |
| 275 | 29.94 | 0.28 |
| 300 | 34.23 | 0.28 |
| 350 | 42.31 | 0.94 |
| 375 | 47.17 | 0.58 |
| 400 | 54.16 | 1.01 |

The polythermal technique enables both a dissolution value (solubility) and re-crystallisation (metastable limit) to be recorded and the metastable limit distributions are shown below in Figure 4. against the extrapolated, best estimated solubility values.

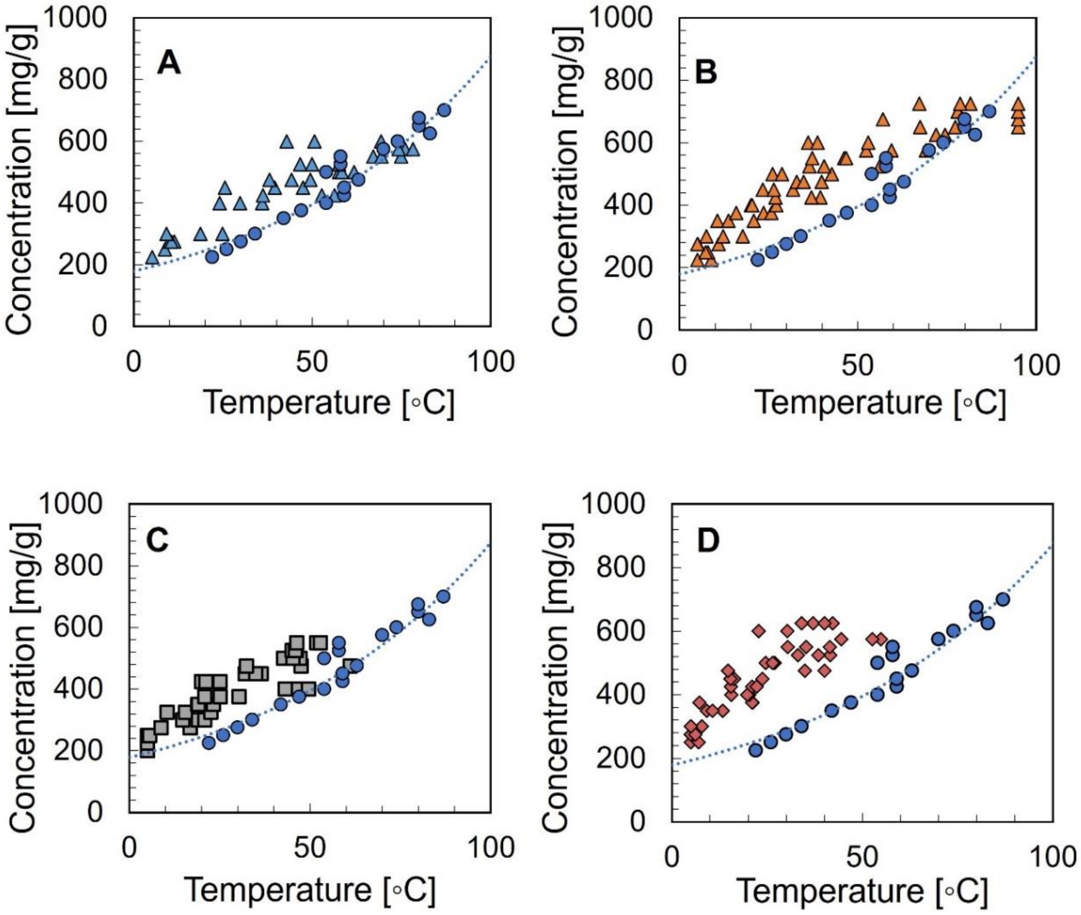


**Figure 4.** The metastable zone width is shown at 4 different cooling rates ranging from 0.1 – 0.5°C/ min. The solubility displayed is the extrapolated 'true equilibrium' solubility. A cooling rate of 0.1°C/ min is shown in A with blue triangles, 0.2°C/ min (B) (orange triangles), 0.3°C/ min (C) (grey squares) and 0.5°C/ min (D) (orange diamonds). As shown, the metastable zone width gets broader with an increased rate of cooling.

As the rate of cooling is increased, the system has less time available to reach a thermodynamic equilibrium, for a nucleus to form, and grow to a detectable size. This is shown when comparing the cooling rates at 0.1 and 0.5°C/ min. With a cooling rate of 0.5°C/ min, there is an onset of primary nucleation much later which results in a broad metastable zone width. Alternatively, a cooling rate of 0.1°C/ min suggests that there is a narrow metastable zone. The significant reduction is therefore expected to be simply representative of the system having enough time to adjust before detection of nucleation which in this case requires multiple successive events. The significant distribution of 'cloud

point' measurements indicates therefore that there is significant stochastic element in these unseeded measurements.

As mentioned in the main text, the induction time data were plotted as probability distributions *P*(*t*) and these were fitted with a Poisson distribution. The raw traces are shown in Figure 5.

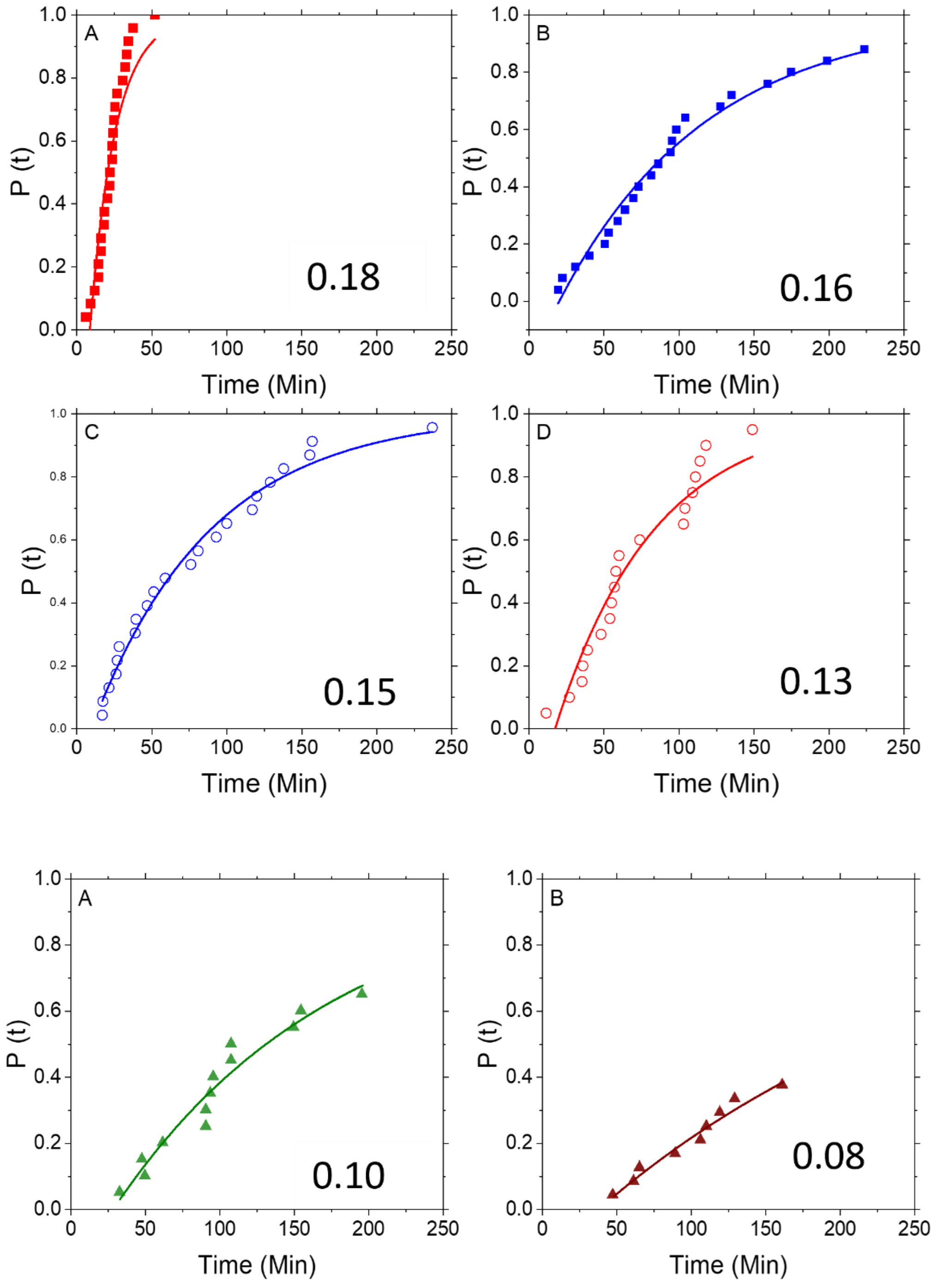


**Figure 5.** Cumulative probability distributions of the stochastic induction time data for the glycine in water system across multiple supersaturations. There is an increase in primary nucleation rate (J)

indicated by the steepness of the slope, with an increase in the supersaturation. The supersaturation is displayed in the bottom right region of each graph.

To visualise this decrease of growth time $t_g$ and corresponding increase in primary nucleation rate $J$ with increasing supersaturation, they have been presented together in Figure 6.

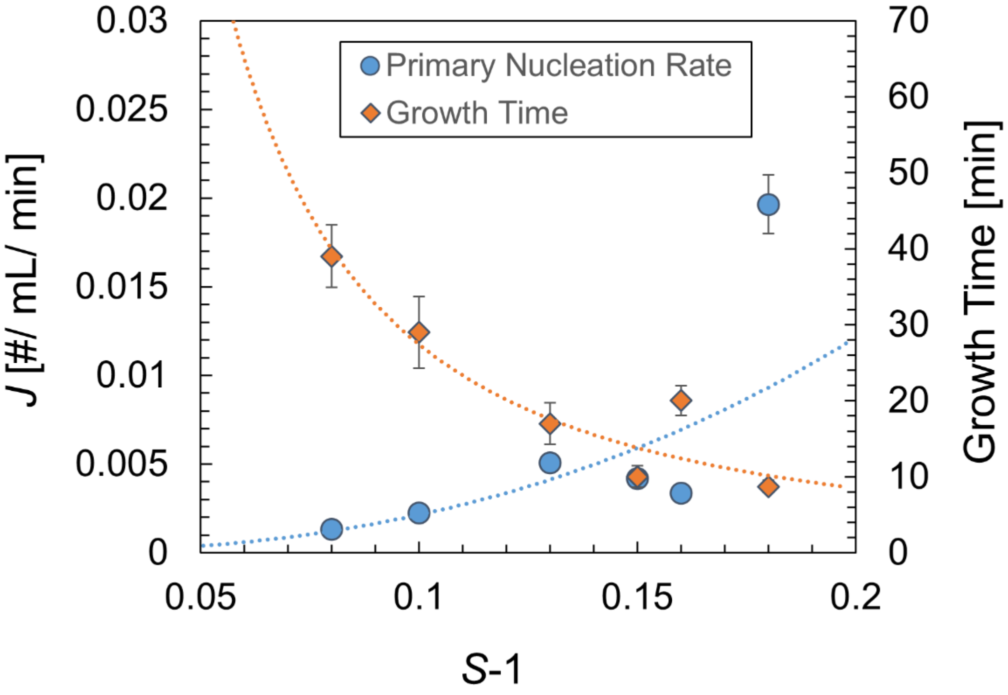


**Figure 6.** The primary nucleation rate $J$ and growth time $t_g$ estimated from the probability distributions vs the corresponding supersaturation. There is an increase in $J$ with an increase supersaturation whilst $t_g$ decreases. The growth time is shown by the orange diamonds and the primary nucleation rate by the blue circles. A power law fit is shown for both and extrapolated to show theoretical trends.

Secondary Nucleation experiments were next conducted in 3 mL volumes under agitation by magnetic stirrer using the Crystalline instrument, obtaining the number of crystals in the observed volume from analysis of images taken with instrument's built-in camera. All measurements were obtained under isothermal conditions at T=25°C, i.e., matching the conditions of the induction time measurements.

The solution, at a given concentration to provide the required supersaturation at T=25°C, was first heated to 55°C and held for a period of 30 minutes to ensure full dissolution. The temperature was then

reduced to 25°C at a rate of 5°C/min to reach the desired supersaturation. At this point, a single crystal seed of a known size (length of 2.5 ± 1 mm) was added to each vial, this procedure is shown in Figure 7. The solution was constantly agitated.

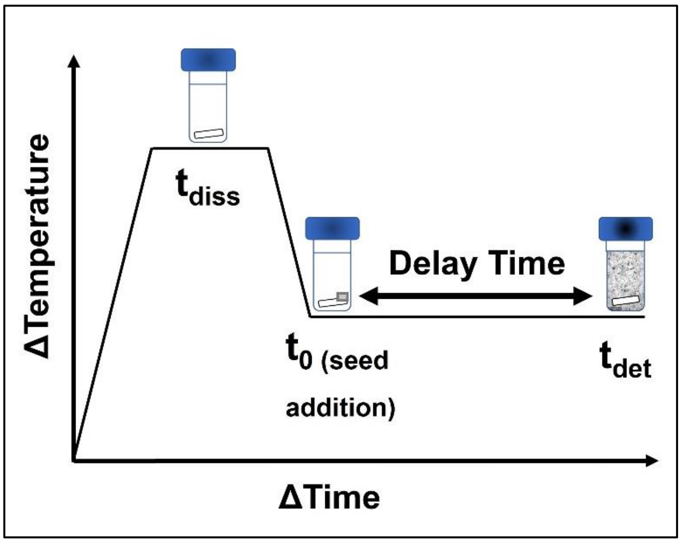


**Figure 7.** A schematic of a typical temperature seeding profile. The temperature is firstly increased to ensure complete dissolution of the solid glycine. This is followed with cooling to the working temperature (and supersaturation) T=25°C. At this point, the seed is added, and the delay time is recorded once secondary nucleation is activated (crystal number >10). The secondary nucleation rate can then be recorded by tracking the change in number over time.

Prior to seed addition, seeds were characterised using optical microscopy and Raman spectroscopy which enabled rapid size and form analysis on a number of seed crystal candidates and provided the opportunity to visually observe the morphological features.

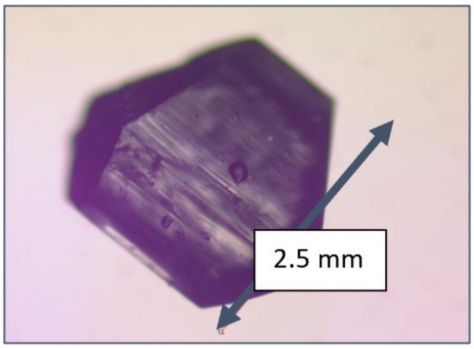


**Figure 7.** An example seed crystal which was characterised using optical microscopy. This seed may be an example of one which would have been subsequently washed and added directly to a Crystalline vial for analysis of the secondary nucleation and crystal growth kinetics.

Raman was used on a selected few seed crystals as it is a non-destructive technique and the specific seed crystals could then be added directly to the Crystalline vials.

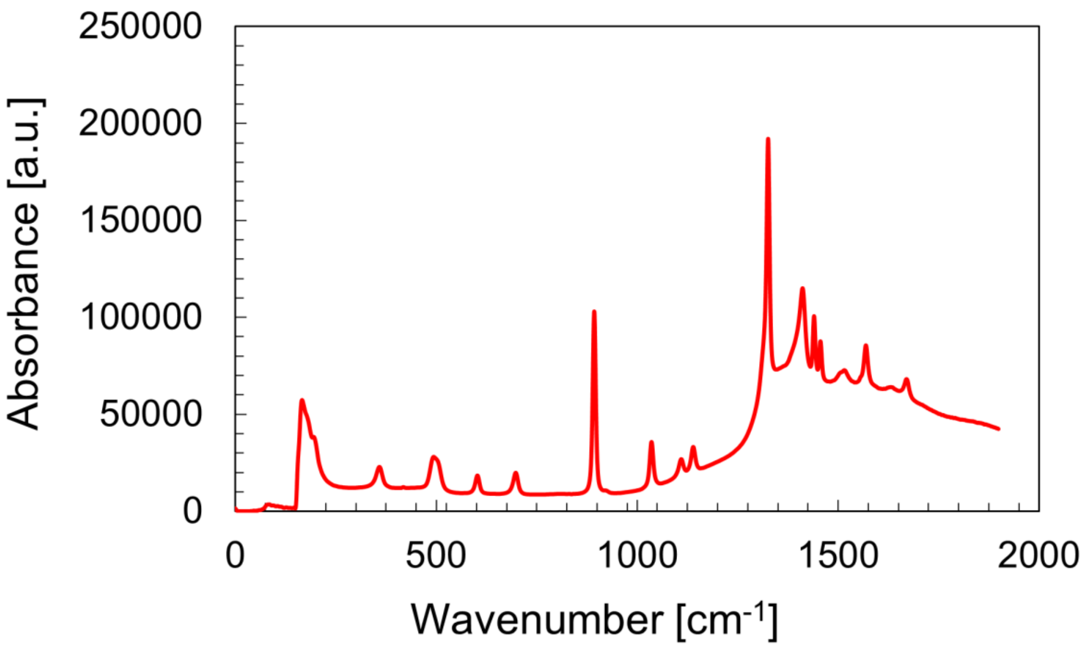


**Figure 8.** Example Raman spectra for α-glycine with characteristic peaks

Once the seed is added, there is a finite amount of time before secondary nucleation is activated, this is known as the delay time. Below in Figure 10, an example is shown displaying a typical seed addition followed by proliferation of crystals from the seed surface.

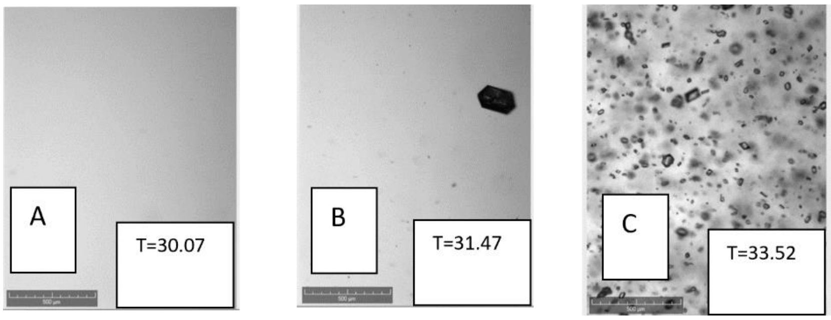


**Figure 10**. Images using the Crystalline instrument, 3 mL vial sample at S=1.17 showing a single seeded secondary nucleation experiment. (A) prior to seeding (B) following seed addition and (C) once secondary nucleation has taken place. Secondary nucleation and growth leads to the number of crystals multiplying rapidly. The time in minutes is shown within each image.

Figure 11 shows the distribution of unseeded, secondary nucleation rates plotted on a linear scale. The vide variation in secondary nucleation rates is likely a result of the variations in the crystal size which initiates secondary nucleation in unseeded vials.

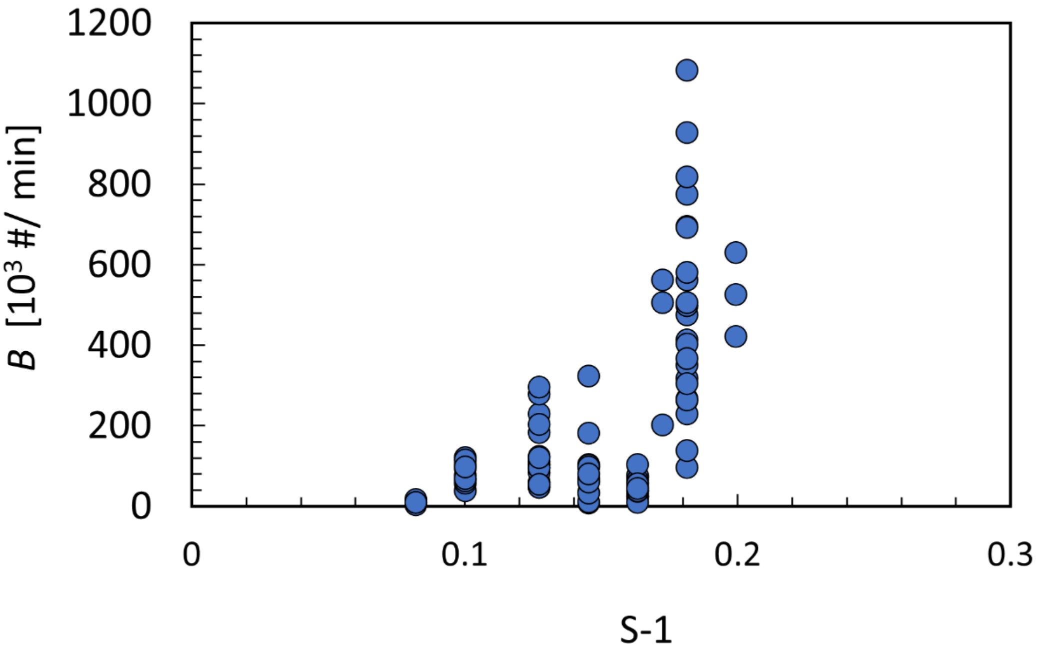


**Figure 11**. The secondary nucleation rate for α-glycine as a function of solution supersaturation expressed as S-1. Secondary nucleation rate determined from unseeded experiments plotted on a linear scale. 8 different supersaturations are shown between S-1 =0.08 and 0.20.
